# Towards understanding a potential *Cryptococcus neoformans* environmental-host transmission route in agricultural settings

**DOI:** 10.1016/j.onehlt.2025.101284

**Published:** 2025-11-20

**Authors:** Monni P. Rapeso, Wynand J. Goosen, Carolina H. Pohl, Olihile M. Sebolai

**Affiliations:** Department of Microbiology and Biochemistry, University of the Free State, Bloemfontein 9301, South Africa

**Keywords:** Agricultural environments, Cattle, *Cryptoccoccus*, Fungal adaptation, One health, Unrecognised transmission route

## Abstract

*Cryptococcus* (*C.*) *neoformans* is a fungal pathogen of global significance. Its ecological niches are well-documented in soils contaminated with bird droppings and decaying vegetation; yet, the full scope of its persistence and transmission within agricultural settings remains underexplored. This perspective paper proposes a potential transmission route involving birds, soil, livestock, particularly cattle, and humans that could sustain the environmental burden of cryptococcal cells on farms. We also highlight the possibility of fungal adaptation after fungal cells cycle in cattle before infecting humans. Finally, we propose some mitigation strategies that might be essential to monitoring and breaking this transmission route.

*Cryptococcus (C.) neoformans* is a basidiomycetous fungal pathogen of global significance, and this is underscored by its inclusion in the first World Health Organisation's Fungal Priority Pathogen List [1], where it is listed as the number one pathogen. This fungus causes cryptococcal meningitis, a life-threatening infection involving inflammation of the central nervous system [2], and is endemic in people living with advanced HIV disease [3], the vast majority of whom are in low and middle-income regions [4]. In these developing parts of the world, three billion rural people live in about 475 million small farm households, working on land plots smaller than two hectares [5].

*C. neoformans* is documented to thrive in a range of environmental reservoirs. The most prominent and well-documented is soil contaminated with bird droppings, particularly from pigeons [6]. Cryptococcal cells have also been isolated from tree hollows and decaying plant matter [7], which are more abundant on farmlands than in urban areas. While not clinically affected by this fungus due to their high internal temperature [8], birds act as major environmental disseminators. Thus, it is foreseeable that they could move between farmlands and urbanised areas, seeding cryptococcal cells across the ecology for potential long-term persistence. Here, the droppings' nitrogenous content and low pH are essential for supporting fungal survival for up to two years [9]. The mechanical disturbance of these droppings could result in aerosolised infectious propagules that can be inhaled and establish a primary lung infection in a susceptible person [6,10].

It is, therefore, plausible that this is also the manner in which livestock may acquire cryptococcal infections, especially considering cattle often inhabit outdoor, soil-rich environments that may be contaminated with bird droppings, including direct inoculation of abraded udder tissue with cryptococcal cells (Fig. 1). The latter is supported by isolated reports documenting *Cryptococcus* infections, which involve respiratory and other disseminated forms, such as mastitis [11,12]. In their review on cryptococcosis in animals and birds, Refai et al. noted that cryptococcosis can present in various animals, and is responsible for severe outbreaks of mastitis in cattle and sporadic cases in buffaloes [11]. For example, Pounden and co-workers documented one of the earliest and most extensive clinical outbreaks of *Cryptococcus* infection affecting 106 cows within a herd of 235 [13]. Notably, the pathogen was isolated from samples even in the absence of visible abnormalities in the mammary gland or milk. Importantly, cryptococcal mastitis in dairy cows is said to be globally distributed [14] and subsequent accounts have expanded the recognised disease spectrum in cattle. To this end, Magalhães and co-workers reported a case of cerebral cryptococcosis in a cow, characterised by cryptococcal masses within the parenchymal organs [12]. Meanwhile, Riet-Correa and co-workers documented cryptococcal meningitis presenting with multifocal neurological deficits [15]. These rare cases confirm that *C. neoformans* can invade the central nervous system and cause a clinically significant disease. The above accounts suggest that cattle may act as incidental hosts in specific environmental contexts, such as farms.

**Fig. 1:**
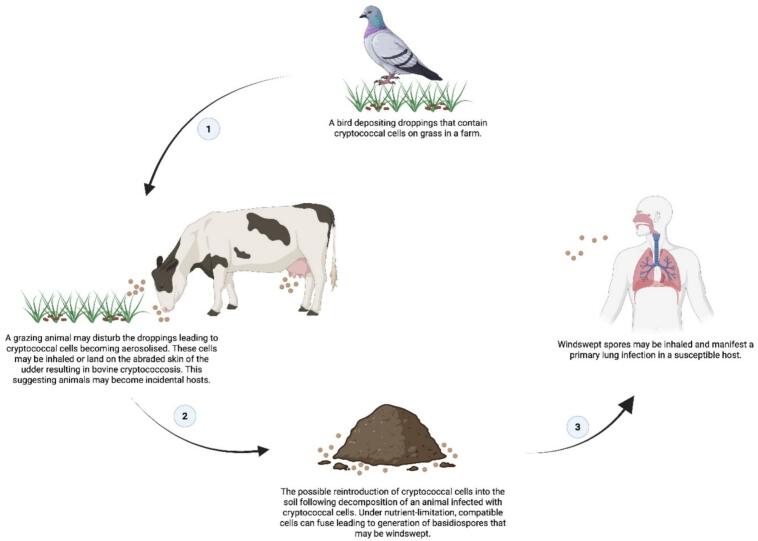
The diagram illustrates how *Cryptococcus neoformans* may persist and recirculate in the farm environment. The infection transmission pathway begins when pigeons shed cryptococcal cells in their droppings, contaminating soil and plant matter. Cattle grazing on contaminated land may become infected through inhalational exposure or direct inoculation of abraded udder tissue. Upon death, decomposing carcasses may, if not incinerated, reintroduce viable fungal cells into the environment. Humans, particularly those living and working on farms, can acquire infection by inhaling aerosolised cells from the same contaminated soil. This transmission route enables long-term persistence of cryptococcal cells in shared environments without requiring direct zoonotic transmission. The image was created using BioRender.com.

We previously proposed that cryptococcal cells, which are generally non-transmissible, likely re-enter the environment when an infected host, such as cattle, dies and decomposes [9]. Thus, the act of burying a diseased animal reintroduces the cells into the environment, and the decomposition process provides nutrient-rich substrates that favour fungal proliferation in an environment that may otherwise be resource-scarce. Interestingly, studies on *Coccidioides* species have revealed that the fungus occupies highly specific ecological niches in arid regions, frequently isolated from soils surrounding decaying mammalian carcasses [16]. Similar to *Cryptococcus*, its cells can be inhaled from the environment and cause infections in both people and animals, such as dogs and cattle, among others [17]. In the context of coccidioidomycosis, dogs residing in endemic zones are known to acquire the infection; however, they are regarded as incidental hosts, as there is no substantial evidence of sustained animal-to-animal transmission [18], although a rare case of transmission to a human via a cat bite has been documented [17]. Such close ecological association parallels what may occur with cryptococcal cells, wherein the remains of infected livestock could enrich the surrounding soil with viable cells.

The above raises the possibility of human inhalational exposure to cryptococcal cells, particularly for farmworkers handling carcasses or in contact with contaminated environments. This unrecognised infection pathway, i.e., bird-livestock-soil-humans, may represent a potential transmission route that allows environmental persistence and may augment the classical bird-soil-human transmission route, thus further contributing to the disease burden. Here, it is pivotal to emphasise that this proposed route is not direct evidence of zoonotic transmission, but instead, this infection pathway represents an environmental spillover, where soil acts as the main reservoir, occasionally amplified or recontaminated through bird droppings or the decomposition of infected animals. To this end, it is prudent to conduct targeted sampling across interconnected sources and perform genotyping in order to assess environmental cycling and determine whether isolates share a common environmental origin or represent independent acquisition events. This approach has been exemplified by the work of Stephen et al. on the outbreak of *C. gattii*, a sister species of *C. neoformans*, which occurred on Vancouver Island, Canada [19]. The outbreak affected animals, such as dogs, which developed cryptococcosis concurrently with humans. The infected animals and humans resided or moved within overlapping ecological zones, suggesting that the infections arose from a common environmental source.

Thus, the cycling of cryptococcal cells in cattle as an incidental host raises intriguing questions. For example, could this enhance the virulence of cryptococcal cells? Experimental evidence suggests that the cycling of cryptococcal cells in holozoic amebae results in amoeba-passaged isolates that exhibit enhanced virulence traits, such as larger capsule size. To illustrate this, Steenburgen and co-workers reported that the passage of an attenuated cryptococcal strain in *Dictyostelium discoideum* resulted in cells with enhanced fungal virulence in a murine infection model [20]. These findings, in part, contributed to the development of the “Amoeboid Predator-Fungal Animal Virulence Hypothesis”, which was proposed by Casadevall and colleagues [21]. However, other studies suggest that not all adaptations may result in cryptococcal cells displaying improved virulence in mammals. This is eloquently demonstrated in the works of Ali et al. and Sauters et al. [22,23] Ali and co-workers showed that cryptococcal cells passaged in *Galleria mellonella* killed fewer mouse macrophages and had less fungal burden in human ex vivo macrophages [22]. Building on this, Sauters et al. [23] argued that there is no correlation between amoeba resistance and increased virulence in murine models, cautioning that while amoeba can shape virulence traits, this may not always translate to increased virulence as predicted by the accidental-pathogen hypothesis by Casadevall and colleagues [21]. The rare traumatic inoculation event where a human subject was bitten by a cat with active coccidioidomycosis provides evidence of an unusual localised disease as the fungus bypassed pulmonary defences [17]. While the fungal cells were directly delivered into the deep tissue, this does not demonstrate increased virulence, as the patient responded to fluconazole therapy and remained asymptomatic at two months after stopping treatment. When considering these studies, it can be rationalised that an infection outcome may vary based on host immune biology, the nature of selection pressure, the route by which infectious propagules are acquired, and the adaptation required. Therefore, one could speculate that in cattle harbouring subclinical cryptococcal infections, the selective pressure from the host immune system could drive its adaptation. However, whether this may be clinically relevant to humans remains unknown, as there is currently no empirical evidence demonstrating that *Cryptococcus* undergoes adaptive changes within cattle to cause severe infections in humans. The latter highlights the need for research into fungal adaptation in livestock and clinical assessment of individuals with cryptococcal infection from farms with known cases of bovine cryptococcosis.

An additional question is whether strains that cycle in cattle can develop the capacity to evolve drug resistance under selective pressure. This is important, as Foy and Trepanier [24] noted that within veterinary medicine, treatment options for systemic mycoses also include the use of antifungal drugs, such as amphotericin B and fluconazole, among others. The overuse of these medicines could desensitise environmental isolates. If true, this raises the urgent concern about environmental isolates acquiring resistance traits before ever reaching a human host, as this has important clinical implications for treatment efficacy and disease outcome. This is a point that Smith and co-workers made, namely that the use of azoles in agriculture to protect crops from fungal diseases could lead to non-responsive clinical strains, as observed in an increase in non-fluconazole susceptibility in Ugandan strains from patients with cryptococcosis [25].

In conclusion, the ecology of *C. neoformans* on agricultural land remains an underinvestigated but potentially critical factor in the persistence and reemergence of human infections. By expanding our lens to include livestock and farm environments, we may uncover hidden reservoirs and novel transmission routes. To this end, this perspective paper aimed to highlight this unrecognised transmission route and encourage targeted investigations crucial for mapping fungal transmission pathways and understanding the dynamics that sustain infection within shared environments. Furthermore, interventional measures must be established to disrupt this potential transmission route, supported by implementation guidelines. This may include the early identification and management of infected animals to prevent environmental shedding. The latter extends to the safe disposal of carcasses, preferably by incineration to inactivate fungal propagules. Efforts can also include monitoring the overuse of azoles in livestock to limit fungal infections. Environmental management may also involve controlling bird roosting and removing bird droppings to reduce fungal reservoirs while maintaining consistent use of personal protective equipment to protect animal handlers on farms. A One Health approach, involving surveillance, genotyping, and data sharing across veterinary and public health sectors, can help track the persistence and cycling of cryptococcal cells within agricultural environments. The above strategies can minimise the opportunities for environmental enrichment and airborne transmission, effectively interrupting the ecological cycle that sustains infections in animals and humans.

## Author contribution statement

O.M.S. conceptualised and designed the paper and managed author contributions. P.M.R. assisted with literature searches and wrote the first draft. W.J.G., C.H.P. and O.M.S. edited the manuscript. All authors read and approved the final manuscript. All authors were responsible for the decision to submit the manuscript.

## Funding information

Olihile M. Sebolai is supported by grants from the 10.13039/501100001321National Research Foundation of South Africa (grant no. 137965), the Poliomyelitis Research Foundation of South Africa (grant no. 23/78), and the Medical Research Council of South Africa (SIR grant). Carolina H. Pohl is supported by the 10.13039/501100001321National Research Foundation of South Africa (grant no. 115566).

## CRediT authorship contribution statement

**Monni P. Rapeso:** Writing – original draft. **Wynand J. Goosen:** Writing – review & editing, Supervision. **Carolina H. Pohl:** Writing – review & editing, Supervision. **Olihile M. Sebolai:** Conceptualization, Writing – review & editing, Supervision.

## Declaration of competing interest

The authors declare the following financial interests/personal relationships which may be considered as potential competing interests:

**Table:** 

Olihile M. Sebolai reports administrative support, article publishing charges, equipment, drugs, or supplies, and statistical analysis were provided by National Research Foundation of South Africa. Olihile M. Sebolai reports administrative support, article publishing charges, equipment, drugs, or supplies, and statistical analysis were provided by South African Medical Research Council. Olihile M. Sebolai reports administrative support, article publishing charges, equipment, drugs, or supplies, and statistical analysis were provided by Poliomyelitis Research Foundation of South Africa. Carolina H. Pohl reports administrative support, article publishing charges, equipment, drugs, or supplies, and statistical analysis were provided by National Research Foundation of South Africa. If there are other authors, they declare that they have no known competing financial interests or personal relationships that could have appeared to influence the work reported in this paper.

## Data Availability

No data was used for the research described in the article.

## References

[1] World Health Organization (2022). https://iris.who.int/bitstream/handle/10665/363682/9789240060241-eng.pdf?sequence=1.

[2] Bicanin T., Harrison T.S. (2004). Cryptococcal meningitis. Br. Med. Bull..

[3] Rajasingham R., Govender N.P., Jordan A. (2022). The global burden of HIV-associated cryptococcal infection in adults in 2020: a modelling analysis. Lancet Infect. Dis..

[4] World Health Organization (2025). https://www.who.int/teams/global-hiv-hepatitis-and-stis-programmes/hiv/strategic-information/hiv-data-and-statistics.

[5] Food and Agriculture Organization (2015). https://www.fao.org/agrifood-economics/publications/detail/en/c/358059/.

[6] Esakkiammal M., Rajakumari D.S. (2025). Pigeons droppings as a Cryptococcus reservoir: a review. Punjab Univ. J. Zool..

[7] Vreulink J.M., Khayhan K., Hagen F. (2017). Presence of pathogenic cryptococci on trees situated in two recreational areas in South Africa. Fungal Ecol..

[8] Johnston S.A., Voelz K., May R.C. (2016). Cryptococcus neoformans thermotolerance to avian body temperature is sufficient for extracellular growth but not intracellular survival in macrophages. Sci. Rep..

[9] Maliehe M., Ntoi M.A., Lahiri S. (2020). Environmental factors that contribute to the maintenance of Cryptococcus neoformans pathogenesis. Microorganisms.

[10] Powell K.E., Dahl B.A., Weeks R.J. (1972). Airborne Cryptococcus neoformans: particles from pigeon extraction compatible with alveolar deposition. J. Infect. Dis..

[11] Refai M.K., El-Hariri Alarousy R. (2017). Cryptococcosis in animals and birds: a review. Eur. J. Acad. Res..

[12] Magalhães G.M., Saut J.E., Beninati T. (2012). Cerebral cryptococcomas in a cow. J. Comp. Pathol..

[13] Pounden W.D., Amberson J.M., Jaegeer R.F. (1952). A severe mastitis problem associated with C. Neoformans in a large dairy herd. Amer. J. Vet. Res..

[14] Rippon J.W. (1982).

[15] Riet-Correa F., Krockenberger M., Dantas A.F. (2011). Bovine cryptococcal meningoencephalitis. J. Vet. Diagn. Invest..

[16] del Rocío Reyes-Montes M., Pérez-Huitrón M.A., Ocaña-Monroy J.L. (2016). The habitat of Coccidioides spp. and the role of animals as reservoirs and disseminators in nature. BMC Infect. Dis..

[17] Gaidici A., Saubolle M.A. (2009). Transmission of coccidioidomycosis to a human via a cat bite. J. Clin. Microbiol..

[18] Graupmann-Kuzma A., Valentine B.A., Shubitz L.F. (2008). Coccidioidomycosis in dogs and cats: a review. J. Am. Anim. Hosp. Assoc..

[19] Stephen C., Lester S., Black W. (2002). Multispecies outbreak of cryptococcosis on southern Vancouver Island, British Columbia. Can. Vet. J.-Rev. Vet. Can..

[20] Steenbergen J.N., Nosanchuk J.D., Malliaris S.D. (2003). Cryptococcus neoformans virulence is enhanced after growth in the genetically malleable host Dictyostelium discoideum. Infect. Immun..

[21] Casadevall A., Fu M.S., Guimaraes A.J. (2019). The ‘amoeboid predator-fungal animal virulence’ hypothesis. J. Fungi.

[22] Ali M.F., Tansie S.M., Shahan J.R. (2020). Serial passage of Cryptococcus neoformans in galleria mellonella results in increased capsule and intracellular replication in hemocytes but not increased resistance to hydrogen peroxide. Pathogens.

[23] Sauters T.J.C., Roth C., Murray D. (2023). Amoeba predation of Cryptococcus: a quantitative and population genomic evaluation of the accidental pathogen hypothesis. PLoS Pathog..

[24] Foy D.S., Trepanier L.A. (2010). Antifungal treatment of small animal veterinary patients. Vet. Clin. N. Am..

[25] Smith K.D., Achan B., Hullsiek K.H. (2015). Increased antifungal drug resistance in clinical isolates of Cryptococcus neoformans in Uganda. J. Antimicrob. Agents.

